# Synthesis Strategies, Optical Mechanisms, and Applications of Dual-Emissive Carbon Dots

**DOI:** 10.3390/nano13212869

**Published:** 2023-10-30

**Authors:** Yuqian Liu, Fangyuan Liang, Jianglei Sun, Ran Sun, Chao Liu, Chao Deng, Farzad Seidi

**Affiliations:** Jiangsu Co-Innovation Center of Efficient Processing and Utilization of Forest Resources and Joint International Research Lab of Lignocellulosic Functional Materials, College of Light Industry and Food Engineering, Nanjing Forestry University, Nanjing 210037, China; fangyuan@njfu.edu.cn (F.L.); sssun@njfu.edu.cn (J.S.); sunran@njfu.edu.cn (R.S.); chaoliulc@njfu.edu.cn (C.L.); cdeng@njfu.edu.cn (C.D.); f_seidi@njfu.edu.cn (F.S.)

**Keywords:** dual-emissive carbon dots, optical mechanism, ratiometric fluorescence, synthesis strategies, white-light-emitting diodes

## Abstract

Tuning the optical properties of carbon dots (CDs) and figuring out the mechanisms underneath the emissive phenomena have been one of the most cutting-edge topics in the development of carbon-based nanomaterials. Dual-emissive CDs possess the intrinsic dual-emission character upon single-wavelength excitation, which significantly benefits their multi-purpose applications. Explosive exploitations of dual-emissive CDs have been reported during the past five years. Nevertheless, there is a lack of a systematic summary of the rising star nanomaterial. In this review, we summarize the synthesis strategies and optical mechanisms of the dual-emissive CDs. The applications in the areas of biosensing, bioimaging, as well as photoelectronic devices are also outlined. The last section presents the main challenges and perspectives in further promoting the development of dual-emissive CDs. By covering the most vital publications, we anticipate that the review is of referential significance for researchers in the synthesis, characterization, and application of dual-emissive CDs.

## 1. Introduction

Multi-emissive materials have never been in such high demand in the areas spanning photoelectronics and biomedical photonics. Among the photoelectronic devices, white-light-emitting diodes (WLEDs) with low energy consumption, long lifetime, and environmental friendliness can be applied in various fields [1,2]. By combining fluorophores covering the entire visible light range, researchers have devised a variety of WLEDs in the past [3,4,5]. In the biomedical area, fluorescent sensors with multi-band emissions, i.e., ratiometric sensors, are appealing due to the merits of excluding interferences from environmental factors (light source power, auto-fluorescence molecules in the matrix, etc.) [6,7]. This self-calibration effect is also vital for ratiometric fluorescent probes applied in bioimaging [8]. For the sake of achieving unique multi-emissive properties, single-emissive fluorophores (molecular dyes [9], semiconductor quantum dots [10], gold nanoclusters [11], upconversion nanoparticles [12], etc.) were usually integrated by either mixing or assembling. For multi-component systems, several imperfections should be addressed in practical applications. For instance, phase separation and color aging are still challenging problems for WLEDs comprised of multiple fluorophores, while the tedious preparation/purification and batch-to-batch variation in relative emission intensity are common for ratiometric fluorescent sensors/probes. These problems prompt researchers to exploit alternatives to the traditional multi-component fluorescent systems. In 2009, Peng’s group reported a Cu-doped InP/ZnSe quantum dot with adjustable dual-emissive property (bandgap emission and Cu dopant emission), opening the prelude of research on “dual-emission in one dot” [13]. Unlike the multi-component fluorescent systems, the relative emission intensity, as well as the emission wavelength of the dual-emissive semiconductor quantum dots, was customized during their synthesis, revealing superior optical consistency in the subsequent applications. Zhang et al. [14] and Peng et al. [15] fabricated WLEDs by the use of dual-emissive Cu:CdS/ZnSe and Mn:ZnCuIn/S quantum dots, respectively. Simultaneously, the ratiometric fluorescent sensings/imagings of organophosphate [16], folic acid [17], metal ions [18,19], and pH [20,21] were achieved using the dual-emissive quantum dots.

Carbon dots (CDs) are zero-dimensional emissive particles with diameters within 10 nm. Due to their appealing properties, including high quantum yield (QY), photo-bleaching resistance, excellent hydrophilicity, satisfying biocompatibility, easy to synthesize, abundant precursor sources, and so forth, CDs have become ideal alternatives to conventional organic dyes or semiconductor quantum dots [22,23]. In 2004, Scrivens’s group first discovered fluorescent carbon nanoparticles during the purification of arc-synthesized single-walled carbon nanotubes [24]. Thereafter, a diverse range of CD preparation methods have been exploited. Overall, CDs can be prepared by “top-down” (arc discharge [25], laser ablation [26], nanometer etching [27], etc.) or “bottom-up” (pyrolyzation [28], hydrothermal/solvothermal [29], microwave assistant pyrolysis [30], etc.) methods, which have been well-documented before. In comparison, the “bottom-up” methods are the most extensively approbatory because of the merits, including (1) large-scale and low-cost CD synthesis is attainable, (2) CDs reveal superior QY and uniformity, and (3) the composition and size of CDs can be tailored by precursor selection or changing the nucleation/growth conditions.

In parallel with the preparation methods, emission color regulation, as well as the mechanism demonstration, of CDs has been vastly investigated during the past decade [31]. Unlike the organic dyes or semiconductor quantum dots, the emission behavior of CDs is complex due to the dependence on the π-conjugated carbon core with sp^2^ hybridized C, molecular state emission, and sp^3^ C dominated surface trap states [32]. Therefore, tuning the CD emission wavelength can be realized by adjusting the size of the π-domain, the abundance/type of molecular emission centers, or surface groups. For CDs with a large π-domain but few molecular emission centers or surface traps, the bandgap emission is dominant, which is similar to semiconductor quantum dots with the quantum size effect. The emission wavelength is thus a function of the CD particle size [33,34]. For CDs with abundant molecular emitters, which might be formed during the dehydration of precursors, the CD emission color depends on the molecules [35]. Due to the diversity of energy levels of the surface groups, various emission traps can be introduced to CDs, giving rise to the emission wavelength tuning of CDs by adjusting their surface chemical property or dispersion conditions (pH, solvent polarity, concentration, etc.) [36,37,38]. Noticeably, heteroatom doping, such as B, N, S, P, and metal ions, is an effective way to modulate the emission wavelength of CDs. Either being embedded in the graphitic carbon core or forming other chemical groups, the heteroatoms are capable of modulating the intrinsic energy levels of CDs or creating new emission centers, causing the emission wavelength shift of CDs [39,40]. In most cases, the emission profile of CDs could be the spectral overlay of more than one emission center with various energy levels. As a consequence, the full width at half maximum (FWHM) values of CDs are usually larger than other fluorophores, and excitation-dependent emission is a common phenomenon for CDs. However, if the multiple emission centers within one dot reveal less effective overlap and, more importantly, fluoresce with single wavelength excitation, the multi-emissive character can be achieved for CDs. During the past five years, a significant number of review articles summarized the synthesis protocols, intrinsic structures, optical mechanisms, and applications of CDs [41,42,43]. However, the review articles focused on the multi-emissive CDs are insufficient. As far as we know, only one paper reviewed the dual-emissive CDs that resulted from the inter-particle interaction and metallic atom doping, as well as their ratiometric sensing applications [44]. Herein, being aware of their fascinating optical properties, we propose to exclusively summarize the dual-emissive CDs from the perspectives of synthesis, emission mechanisms, and applications.

## 2. Synthesis Strategies for Dual-Emissive CDs

**Figure 1:**
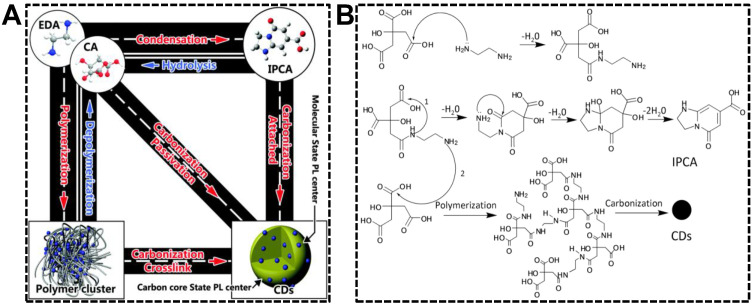
(**A**) Translational relationship of the products in the upon hydrothermal treatment of citric acid and ethylenediamine. (**B**) Forming mechanism of the molecule (IPCA), polymer cluster, and CDs. Reproduced with permission [46]. Copyright 2015, The Royal Society of Chemistry.

Synthesis strategy, the precursors and reaction conditions in particular, produces the most remarkable impacts on the properties of nanomaterials. CDs are not exceptional. It is well known that CDs’ chemical and optical properties are established as prepared and can rarely be changed in subsequent treatments. Optimizing the synthesis strategy is thus the first choice to acquire CDs with desirable QYs or emission wavelength. As far as we know, dual-emissive CDs have not been obtained from the “top-down” approaches. Therefore, we dedicatedly discuss the “bottom-up” approaches in preparing dual-emissive CDs in this section. From the CD formation mechanism during the hydrothermal/solvothermal treatments of precursors, the products, as well as the degree of graphitization of CDs, rely heavily on the treatment condition and the precursor composition [45]. Yang’s group systematically investigated the chemical procedure of CD synthesis by pyrolyzing citric acid and ethylenediamine [46]. As shown in Figure 1, CDs are achievable upon the precursors that are successively condensed/polymerized and carbonized in the system. In case the intermediate molecule (precursor dimer) or polymer possesses the conjugated structure, CDs are not the sole fluorophore in the systems that have not experienced harsh carbonization conditions. Hence, controllable carbonization is an effective strategy for preparing CDs exhibiting emissions from carbon skeleton and molecular/polymeric centers.

Except for regulation of the synthesis conditions, precursors with specific structures can directly or indirectly render CDs with a new emission band beyond the carbon core emission. The optical and structural properties of precursors could be “semi-reserved” in CDs due to incomplete carbonization, leading to the introduction of molecular emission centers (direct manner) or cross-linking among CDs for the generation of new emission bands (indirect manner). Doping with heteroatoms is also a feasible strategy to prepare dual-emissive CDs by introducing new surface properties, creating trap states, or causing electronic interactions among carbon atoms with the neighboring dopant atoms [47,48].

### 2.1. Controllable Carbonization

**Figure 2:**
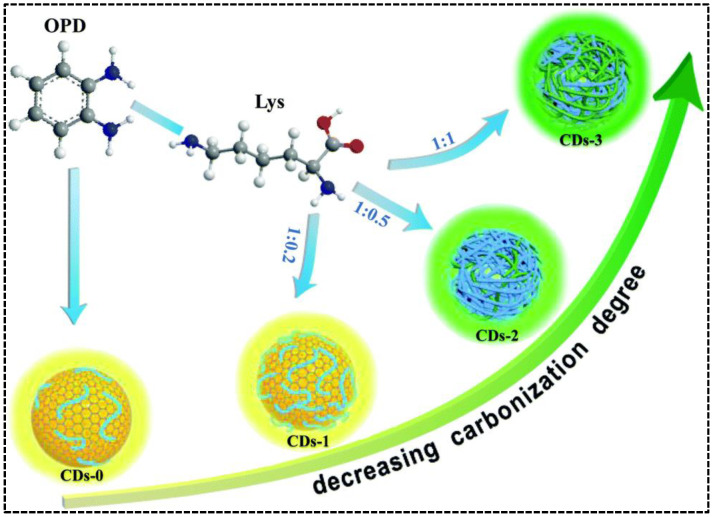
Schematic representation of the fabrication of the dual-emissive CDs with OPD and Lys as precursors. Reproduced with permission [49]. Copyright 2020, The Royal Society of Chemistry.

By carefully adjusting the polymerization and carbonization procedure of precursors o-phenylenediamine (OPD) and lysine (Lys) during hydrothermal treatment, Chen’s group synthesized CDs with tunable blue and green emissions [49]. In the absence of Lys, self-polymerization of OPD molecules and carbonization of OPD polymer chains resulted in the formation of blue/green emissive CDs. Adding Lys can suppress the carbonization of the OPD polymer but enhance the self-polymerization of OPD molecules, leading to improved blue emission. Simultaneously, the green emissive OPD-Lys co-polymer endowed the CDs with a new green emission. As such, the relative green-to-blue emission intensity was adjustable by regulating the mass ratio of OPD and Lys (Figure 2). Unlike the precursor composition-regulated carbonization, Kainth et al. utilized different oxidation and dehydration capacities of mineral acids to regulate the carbonization degree of precursor [50]. In their work, the dual-emissive CDs were synthesized from sucrose, which was acid-oxidized by the mixture of H_2_SO_4_ and H_3_PO_4_ with the assistance of a microwave. The green-to-blue intensity ratio of the CDs was influenced by the ratio of acids because H_2_SO_4_ and H_3_PO_4_ played independent roles. H_3_PO_4_ promoted the carbonization at slower kinetics to produce green-emissive surface defects, whereas H_2_SO_4_ caused the blue emissions due to its stronger oxidizing ability to oxidize C-H into O=C-H or C-OH and its dehydrating property to generate unsaturation from C-C. The carbonization process could be enhanced if the concentration of H_2_SO_4_ was too high, leading to the elimination of surface or edge functional group-induced longer wavelength emission in the CDs. Therefore, fixation of a particular molar ratio of H_2_SO_4_ and H_3_PO_4_ (1:2) was necessary to achieve the CDs with appropriate dual emissions. As the most facile approach, adjusting the treatment temperature and time on the precursor(s) is adopted by Liu et al. [51]. They synthesized green/red dual-emissive CDs using 2,5-diaminotoluene sulfate and ethanol and found that the intensity of red emission was dependent on the solvothermal conditions (time/temperature/solvent volume). The optimizable green-to-red emission ratio indicated that external conditions can directly impact the carbonization degree, as well as the optical properties of CDs.

### 2.2. Semi-Reservation of Precursor Structure

**Figure 3:**
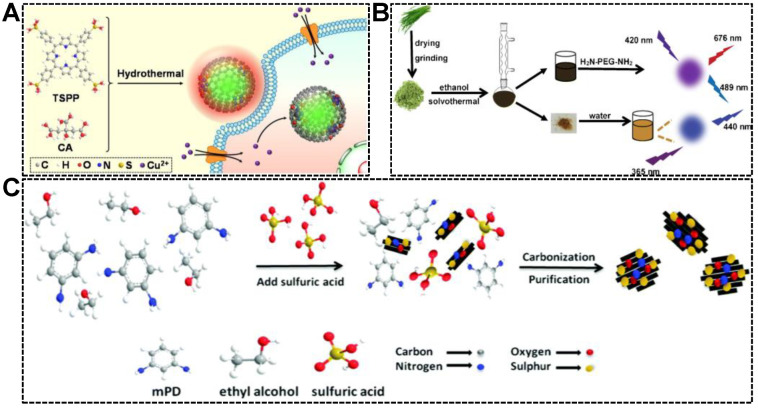
(**A**) Illustration of the synthesis of dual-emissive CDs from citric acid (CA) and TSPP, and the application for intracellular Cu^2+^ detection. Reproduced with permission [57]. Copyright 2021, Elsevier B.V. (**B**) Procedures for the one-pot solvothermal synthesis of dual-emissive and single-emissive CDs from leek. Reproduced with permission [58]. Copyright 2020, Springer Nature. (**C**) Schematic of the preparation of the dual-emissive CDs from mPD and ethanol. Reproduced with permission [60]. Copyright 2017, The Royal Society of Chemistry.

It has been well-documented that the chemical properties of CDs can be tailored by precursor engineering. For instance, folic acid-derived CDs were capable of targeting and imaging cancer cells, of which the folic acid receptor was overexpressed [52,53]. CDs derived from metal chelators were useful for the detection of metal ions, which were complexed by the CDs and caused their fluorescence quenching [54,55,56]. Inspired by the property customizability of CDs, the dual-emissive character can be achieved by the use of precursors with π-conjugated structures. Porphyrins are a family of macrocycle compounds with an extended π-electron system. Our group synthesized a series of green/red dual-emissive CDs using 5,10,15,20-tetrakis(4-sulfophenyl)porphyrin (TSPP) and citric acid. We deduced that the green emission originated from the carbon core, while the red emission was linked to the partially carbonized TSPP residues. As shown in Figure 3A, by adjusting the precursor ratio (citric acid-to-TSPP), the relative green-to-red emission intensity was customizable [57]. Similar works were reported by Shi’s group and Guo’s group (Figure 3B) [58,59]. Dual-emissive CDs were solvothermally synthesized from leek and cabbage, respectively. As is well known, a certain amount of pigments in biomass resources are porphyrin derivatives, which are considered responsible for introducing red emission to CDs. Except for porphyrin-based precursors, other types of molecules with aromatic structures were frequently adopted for the synthesis of dual-emissive CDs. The aromatic precursors carrying N or S elements can produce CDs simultaneously emitting longer wavelength fluorescence (yellow or red emission) and shorter wavelength fluorescence (blue or green). As shown in Figure 3C, m-phenylenediamine (mPD) and ethanol were subjected to oxidation and dehydration in the presence of concentrated H_2_SO_4_, forming CD particles with multiple emission centers [60]. The emissions centered at 360 nm and 550 nm were attributed to the effects of the intrinsic electronic conjugate structure formed by aromatic precursor and surface functional groups, respectively. Using 2,3-diaminobenzoic acid hydrochloride as the precursor, Liu et al. simultaneously synthesized two kinds of dual-emissive CDs, which can emit red/yellow fluorescence and red/orange fluorescence [61]. In their work, the conjugated structure of carbon source was claimed to play an essential role in producing the dual-emission of CDs.

**Figure 4:**
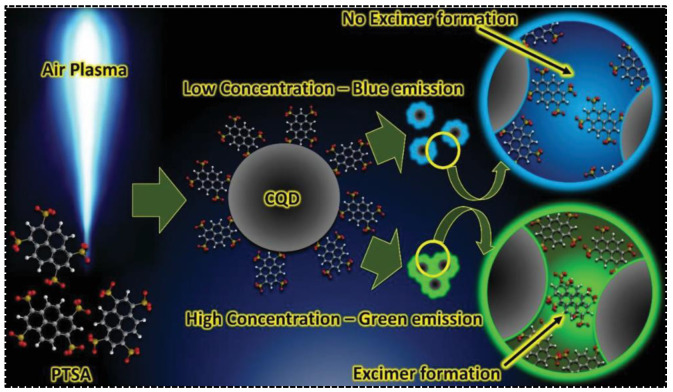
Synthesis and dual-emission mechanisms of CDs prepared by air plasma technique. Reproduced with permission [62]. Copyright 2021, Wiley-VCH GmbH.

In addition to being carbonized and embedded into the carbon skeleton to directly generate one of the emission bands in dual-emissive CDs, the aromatic residues were also evidenced to be modified on the surface of CDs, leading to inter-particle interaction. Additional emission peaks thus arose due to the electronic coupling. 1,3,6,8-Pyrenetetrasulfonic acid contains a large aromatic plane that can be used for the synthesis of CDs with appealing properties. Being served as the precursor, Jainth et al., synthesized blue/green CDs via the atmospheric pressure air plasma treatment [62]. They found that the emission character of CDs was concentration-dependent, exhibiting purple emission at low CD concentrations but green emission at high CD concentrations. The dual-emission was achievable at a CD concentration of at least 1 g/L. The uncarbonized PTSA molecule on the surface of CDs was considered pivotal for green emission. As shown in Figure 4, the green emission was caused by the formation of PTSA dimers driven by π-π interactions. As a consequence, the green emission was switchable by tuning the concentration of CDs. The inter-particle interaction-induced additional emission bands are more commonly seen in those CDs with solid-state fluorescence or phosphorescence. For instance, Yang’s group synthesized dual-emissive CDs with p-aminosalicylic acid and citric acid [63]. The CDs were featured with blue and red emissions in the solid state. The red fluorescence was ascribed to the supramolecular cross-linking between adjacent particles. The surface groups, determined by the precursor type, were crucial for providing the driving forces (π-π interaction, H-bond) and intermediating the supramolecular cross-linking.

### 2.3. Heteroatom Doping Effect

Heteroatom doping has been vastly adopted to modulate fluorescent properties and is considered the most promising engineering approach to produce highly fluorescent CDs. The doping methods, advantages compared to the undoped CDs, as well as applications of doped CDs have been systematically reviewed recently [64,65,66]. Herein, the preparations of unconventional dual-emissive CDs via heteroatom doping strategy will be focused. Esranur et al. synthesized dual-emissive CDs from 3-aminophenylboronic acid (APBA) and boric acid (BA) [67]. The dual-emissive feature of CDs was available only in the presence of high BA amounts. They evidenced that boron was capable of penetrating the CD lattice. Therefore, as the amount of BA increased, the structure of CDs became different, and boron-based energy transitions emerged, giving rise to the dual-emissive property of CDs. Duan’s group prepared orange/red dual-emissive CDs using OPD as the precursor and Al(NO_3_)_3_·9H_2_O as an assistant [68]. Graphitic N appeared in the CD structure (confirmed by the XPS measurements), contributing to the emergence of an additional emission band beyond the intrinsic CD emission.

This section discusses the strategies for producing dual-emissive CDs from different perspectives demonstrated in the published articles. However, in most cases, the dual-emission of CDs is a combinational effect of the mentioned factors. It is noticed that nearly all the precursors used for the synthesis of dual-emissive CDs contain at least one kind of heteroatom, such as S, N, or B. Either being embedded into the graphitic core or forming dangling chemical groups, the heteroatoms were inclined to generate new emission centers in CDs. In addition, precursors with π-domains were more likely to be used in the synthesis of dual-emissive CDs. In comparison to the chain compounds, aromatic structures were, in fact, more stable during the carbonization process due to π-π stacking, allowing the formation of molecular state emissions. Finally, the degree of carbonization produced the most significant impact, which was dominant for bandgap emission or other types of luminescence. Therefore, the unique optical property was achievable only by carefully controlling the carbonization process for all of the dual-emissive CDs.

Another critical issue to be emphasized is that adequate confirmation of the dual-emissive property of CDs is necessary based on the thorough isolation of CDs in the synthesis products. As a common phenomenon for CDs prepared via the bottom-up approaches, the emissive molecular intermediates can significantly impact CDs’ fluorescence. Using citric acid and urea as the typical precursors, Kasprzyk et al. presented molecular insights into the fluorescence of CDs synthesized under different conditions [69]. By sufficient dialysis and analyzing the chemical structures in and out of the dialysis bags, they found that citrazinic acid and 4-hydroxy-1*H*-pyrrolo[3,4-*c*]pyridine-1,3,6(2*H*,5*H*)-trione (HPPT) were responsible for the emission colors, as well as the high quantum yields of blue-emissive CDs (synthesized in sealed reactors) and green-emissive CDs (synthesized without solvent), respectively. As for dual-emissive CDs, researchers should pay more attention to the discrimination of the actual “two-emission in one” CDs and the physical mixtures of CDs/molecular emitter. By the use of glutathione dissolved in formamide, Macairan et al., synthesized the “CDs” with blue and red emissions and investigated their optical mechanism [70]. However, in an updated research, Ganjkhanlou et al., revealed that the emissions at different wavelengths originated from a mixture of physically separate compounds but not the sole CDs [71]. The compounds were identified as blue-emissive CDs and red-emissive porphyrin derivatives, which were separable by adding kaolinite and HCl. Therefore, robust verification of fluorescence behavior is an essential task for the synthesis of dual-emissive CDs.

## 3. Optical Mechanisms of the Dual-Emissive CDs

Different from semiconductor quantum dots, the luminescence mechanism was elaborated decades ago [72]. The optical mechanisms of CDs are far more complicated due to their structural heterogeneity [73,74]. For dual-emissive CDs, the existence of more than one type of emission center is inevitable. Nevertheless, whether the emission centers can be irradiated simultaneously at a single wavelength makes the optical mechanisms of dual-emissive CDs quite different. As is noticed, a significant amount of work investigated the origins of emissions at different wavelengths. However, the relationships between different emission centers were not elaborated. One possible reason is that the transitions of electrons confined in the carbon core and other emission centers (molecules/dopants/functional groups) can occur simultaneously upon the single wavelength excitation, i.e., the emission centers are independent. For the dual-emissive CDs in such a luminescent mechanism, there are supposed to be no electronic or energy interactions among the emission centers. The other possibility lies in the technical hurdles preventing the revelation of a more exact optical mechanism of the dual-emissive CDs. In spite of this, there are some great attempts to provide insight into luminescent mechanisms in their works.

### 3.1. Electron Transfer

Electron transfer has been a widely known mechanism to tune the emission intensity of fluorophores in sensory systems or realize the photovoltaic conversions in LEDs and solar cells [75,76,77]. In CDs, the intra-particle electron transfer was also proven to be responsible for the generation of emission bands beyond bandgap emission. For instance, the CDs prepared from citric acid and TSPP were proven to emit dual emissions due to the intra-particle electron transfer mechanism [57]. From the cyclic voltammetry curve of the CDs, the energy levels for the bandgap and molecular center were calculated. The recombination of photo-excited electrons on the lowest unoccupied molecular orbital (LUMO) and holes on the highest occupied molecular orbital (HOMO) leads to a radiative green emission. Due to the presence of semi-reserved precursors, a portion of the excited electrons were transferred to the LUMO of the TSPP residue, leading to the recombination of excitons with lower energy and the release of photons with wavelengths around 682 nm.

**Figure 5:**
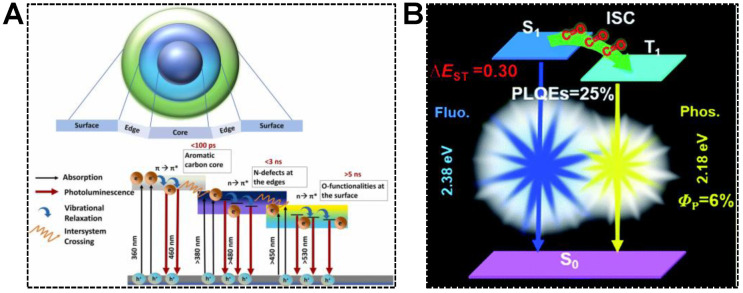
(**A**) Illustration of the CD structure and various radiative pathways responsible for the dual emissions. Reproduced with permission [78]. Copyright 2022, Elsevier Ltd. (**B**) A schematic diagram showing the energy-level diagrams of the relevant photophysical processes of the fluorescence-phosphorescence CDs. Reproduced with permission [79]. Copyright 2019, The Royal Society of Chemistry.

In addition to molecular emission centers, the chemical group, such as carbonyl, is capable of accepting excited electrons in the carbon core. Dual emissions of CDs are thereby achieved in the mechanism of electron transfer, namely, inter-system crossing (ISC). For instance, Chowdhury’s group synthesized dual-emissive CDs using betalain [78]. They concluded that the dual-emissive nature of the CDs was caused by the radiative recombination of excitons in the aromatic carbon core and functional groups attached to the CD surface. As shown in Figure 5A, the mechanism for the emergence of dual-emission bands was proposed as (1) the photoexcited electrons were capable of undergoing synergistic hybridization via π→π* transitions of sp^2^ carbon core to n→π* transitions of the groups located at the edges, (2) the electrons were then relaxed to the C=O energy levels by non-radiative pathway, (3) the electrons were reactivated via direct recombination or ISC, followed by the vibrational relaxation and the generations of dual-emission peaks. Similar findings were reported by Yuan et al. [79]. In the blue/yellow fluorescence/phosphorescence dual-emissive CDs, the carbonyl groups were evidenced to enhance the ISC process and induce intermolecular electronic coupling, resulting in bright yellow phosphorescence (Figure 5B). Quantitatively, the ISC rate (*κ*_isc_) was 1.6 × 10^7^, which was comparable to the corresponding radiative decay rate (*κ*_F_ = 6.6 × 10^7^). The efficient ISC process enabled the production of abundant triplet electrons responsible for intense phosphorescence.

### 3.2. Energy Transfer

**Figure 6:**
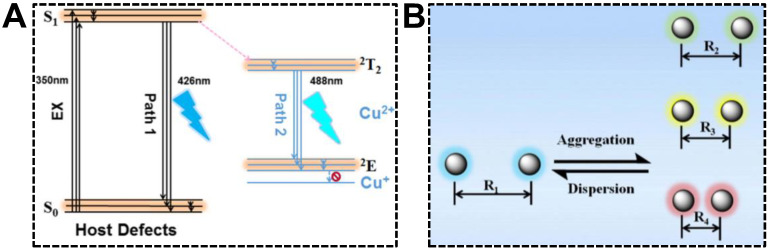
(**A**) Optical mechanism of the Cu(II)-doped dual-emissive CDs. Reproduced with permission [80]. Copyright 2018, American Chemical Society. (**B**) Schematic of the FRET mechanism for the dual-emissive CDs in different solvents. Reproduced with permission [81]. Copyright 2019, Elsevier Inc.

Being served as the dopant, Cu(II) can induce the intra-particle energy transfer in CDs, offering the dual-emission property. Tan’s group investigated the optical mechanism of Cu(II) dopant for the first time using the CDs synthesized with 1-(2-pyridylazo)-2-naphthol (PAN) and CuCl_2_ [80]. The structural and optical characterizations indicated that the aromatic ring ligand in CDs chelated with Cu(II) enhanced energy transfer from the CD host lattice to the Cu(II) energy level. The proposed dual-emission mechanism can be seen in Figure 6A. The primary origin of dual emissions was the defects in CDs. The bandgap of CD host defects located in PAN resulted in the emission centered at 426 nm. In addition, the Cu(II) dopant provided the other energy state (d-d orbital), of which the transition of electrons at the valence band occurred easily. Moreover, the chelation of unpaired electrons on the CD surface and Cu(II) promoted the energy transfer from defects to dopants. The relaxation of electrons in Cu(II) dopant thus provided a new emission at 488 nm. Wu et al. reported that the inter-particle Föster resonance energy transfer (FRET) could render CDs with the dual-emission property as well [81]. The dual-emissive CDs were synthesized from citric acid and formamide and exhibited an incredible solvatochromism phenomenon. In non-aqueous solutions, CDs were able to emit blue and red fluorescence, causing the emission color to turn from red to green, along with the decrement in solvent polarity. The unique solvatochromism phenomenon set the basis for sensing water content in ethanol, which will be summarized in the last section. With respect to the underlying mechanism, it was speculated that hydrogen interactions between CDs and solvents produced impacts on the dispersion states of CDs. According to the FRET principle, energy could be transferred from the donor CD to the neighboring acceptor CDs in a nonradiative process as the distance between the nanoparticles was shorter than Föster radius (*R*_0_) (Figure 6B). As such, the blue emission of CDs served as the light source to excite adjacent CDs, giving rise to the red emission.

### 3.3. Other Mechanisms

Except for the typical electron transfer and energy transfer, other mechanisms responsible for the dual-emissive property of CDs can be occasionally noticed. Among these, achieving an additional emission band via the aggregation-induced emission (AIE) mechanism is a feasible approach. Unlike traditional fluorophores, which are inclined to be quenched at their aggregation state, AIE-active materials show intense fluorescence or phosphorescence upon aggregation [82,83]. Using trimellitic acid as the precursor, Jiang et al. synthesized dual-emissive CDs with blue fluorescence and yellow room-temperature phosphorescence (RTP) [84]. The trimellitic acid precursor transformed into dual-emissive CDs with a large π-domain by dehydration and carbonization. The π-domain, on the one hand, was the origin of the blue fluorescence of CDs, and on the other hand, intermediated the aggregation of CDs in poor solvent or at their solid state by π-π stacking. The aggregation of CDs was considered to stabilize the T_1_ excited state and, more importantly, form another triplet excited state (T_1_*) by delocalization of several T_1_ emission moieties. The yellow phosphorescence was thus produced as the relaxation of T_1_* excitons. Another example of the AIE-induced additional emission can be found in the aforementioned PTSA-derived dual-emissive CDs [62]. The stacking of uncarbonized PTSA molecules resulted in the emergence of green emission. Except for AIE, supramolecular cross-linking among CD particles was proven to contribute to the narrow bandgap emission of CDs [63]. In aqueous solutions, the narrow bandgap fluorophores on CDs cannot fluoresce due to the molecular rotations and vibrations, which resulted in continual energy loss by the nonradiative pathway. However, at the solid state, the supramolecular cross-linking effect enhanced the intensity of narrow bandgap emissions, achieving the dual-emission capacity of CDs.

## 4. Applications for the Dual-Emissive CDs

Benefiting from their facile synthesis and nontrivial optical properties, the dual-emissive CDs are extensively applied to constructions of, but are not limited to, WLEDs and ratiometric fluorescent sensory/imaging systems. In this section, the applications of dual-emissive CDs will be counted. Among these, the signal transduction mechanisms for target sensing will be involved as well.

### 4.1. Constructions of Single-Component WLEDs

Towards the aim of developing efficient dual fluorescence/phosphorescence CDs for achieving single-component WLEDs, Fan’s group first realized the synthesis of blue/yellow fluorescence/phosphorescence CDs using g-C3N4 as the precursor [79]. The CDs revealed an overall photoluminescence quantum efficiency of 25% and relatively bright yellow phosphorescence with a quantum efficiency of 6%. The efficient blue/yellow fluorescence/phosphorescence was compelling for their applications in high-performance single-component WLEDs. A UV-pumped WLED was thus fabricated by coating dual-emissive CDs onto the surface of a UV-LED. The LED exhibited bright white emission under a 20 mA forward bias current, while its electroluminescence spectrum covered a broad spectral region from 380 to 780 nm with two distinct peaks at 450 nm (fluorescence) and 570 nm (phosphorescence). The CIE chromaticity coordinates of the device were determined as (0.35, 0.39), which gave a correlated color temperature of 4935 K. For further improving the efficiency of single-component WLEDs, Tan’s group synthesized the blue/green fluorescence/phosphorescence CDs using lycorine hydrochloride and boric acid as precursors. [85] Since the CDs were embedded in the boric acid matrix, the aggregation-induced quenching in the solid state of the CDs was effectively avoided. As a consequence, the CDs revealed an extraordinarily high overall quantum efficiency of 46% and phosphorescence efficiency of 30%. Finally, the CDs were applied for the fabrication of a single-component WLED, which possessed favorable white light characteristics with a color rendering index of 82.

### 4.2. Ratiometric Fluorescent Sensing

The most apparent advantage of dual-emissive CDs is their feasibility to simultaneously provide a background fluorescence and responsive signal in one nanosensor without any post-treatment. The background fluorescence plays a vital role in providing the self-calibration effect, as emphasized in Section 1. Moreover, emission color change brought by variation in the relative emission ratio of the sensor can greatly benefit the visualized detection [86,87]. In comparison to the ratiometric fluorescent sensors constructed by assembling/linking multiple single-emissive fluorophores [88,89,90], the sensors based on dual-emissive CDs are obviously advantageous in terms of cost and labor effectiveness.

**Figure 7:**
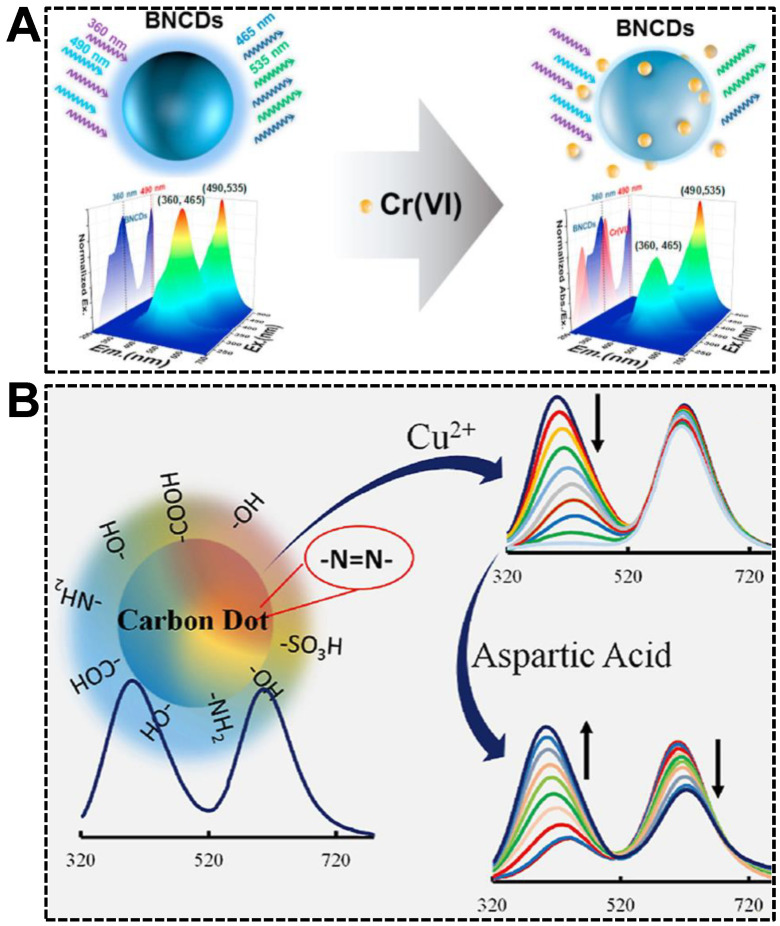
(**A**) Illustration of dual-emissive CDs-based ratiometric fluorescent sensor for the detection of Cr(VI). Reproduced with permission [93]. Copyright 2021, Elsevier Ltd. (**B**) Detections of Cu(II) and aspartic acid. Reproduced with permission [96]. Copyright 2020, Elsevier B.V.

Relying on the avid bindings between electron-deficient metal ions and electron-rich atoms such as O, N, and S, which are usually present on the surface of CDs, in combination with the fluorescence quenching effect of metal ions, dual-emissive CDs-based sensors for ratiometric fluorescent detection of metal ions sprang up recently. Among the metal ions, transition metals are the most frequently reported species due to their easiness to be complexed and efficient electron-withdrawing ability [91,92]. Both are stemmed from their empty d-orbital. For instance, Jia et al. reported a versatile sensing platform for Cr(VI) by the use of B, N co-doped dual-emissive CDs [93]. As shown in Figure 7A, the 3D fluorescence spectrum revealed that the CDs exhibited dual emissions centered at (EX wavelength, EM wavelength) = (360, 465) nm and (490, 535) nm, respectively. In the presence of Cr(VI), the blue emission was quenched in the mechanism of the inner filter effect (IFE). In contrast, the green emission remained unchanged. The sensor exhibited excellent sensitivity (LOD: 0.41 μM) and anti-interference capability, facilitating the sensing in real samples such as lake water, textile leachate, and soil extract. Alterations in copper homeostasis are confirmed to be closely correlated with genetic or neurodegenerative diseases. Therefore, quantifying Cu(II) accurately in complex biofluids, the serum, for instance, with the ratiometric fluorescent sensors, attracted significant attention [94,95]. Based on the specific quenching of the blue emission in dual-emissive CDs by Cu(II), Barati’s group achieved the ratiometric fluorescent detection of Cu(II) with a sensitivity of 7 nM (Figure 7B) [96]. Moreover, aspartic acid could restore the quenched fluorescence of the CDs-Cu(II) system at pH 4.0, allowing the detection of aspartic acid.

**Figure 8:**
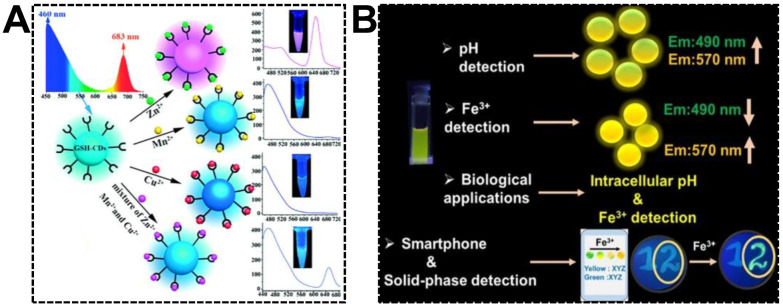
(**A**) Synthesis of GSH-derived dual-emissive CDs using the hydrothermal approach and based on the mechanism of fluorescence discoloration to distinguish Zn(II), Mn(II), and Cu(II) separately or simultaneously. Reproduced with permission [97]. Copyright 2018, Wiley-VCH Verlag GmbH & Co. KGaA, Weinheim. (**B**) Schematic representation of the synthesis of dual-emissive CDs and their various applications in pH and Fe(III) detections. Reproduced with permission [98]. Copyright 2022, American Chemical Society.

Multiplexed detection requires different signal responsiveness in the presence of multiple targets. For dual-emissive CDs, emission peaks at different wavelengths might show varying optical behaviors, which is of great significance in exploring multiplexed ratiometric sensors. By noticing the various fluorescent responsiveness of glutathione-derived dual-emissive CDs towards Zn(II), Mn(II), and Cu(II), Chai’s group first devised the ratiometric fluorescent sensor for individual or simultaneous detections of these metal ions [97]. As shown in Figure 8A, due to the selective quenching of blue or red emission, the color of the dual-emissive CDs transformed from cyan to pink and cyan to blue upon the additions of Zn(II) and Mn(II)/Cu(II), respectively. A natter blue emission color was generated while the CDs were exposed to the three ions. Relying on its capability to exclude the interferences of other metal ions, the dual-emissive CDs were finally challenged to discriminate Zn(II), Mn(II), and Cu(II) in environmental and biological samples. Nandi et al. reported the detections of pH (hydrogen ion) and Fe(III) using dual-emissive CDs synthesized from 3,4-diaminobenzoic acid and hydrazine hydrate (Figure 8B) [98]. It was found that the relative emission intensity of the bands at 490 nm and 570 nm was evidently dependent on the pH of the medium. At the pH range of 2.0–7.6, the CDs can be served as a robust pH sensor. However, in the presence of trace amounts of Fe(III), the emission at 490 nm quenched drastically. Instead, the 570 nm emission was enhanced. The increased yellow-to-blue emission intensity ratio gave rise to the color change from light green to bright yellow with increasing Fe(III) amounts. It was evidenced that the complexation between CDs and Fe(III) and the accompanied aggregation was the dominant reason for the ratiometric fluorescent response. For the sake of on-site Fe(III) detection, a smartphone-based sensing platform was devised by immobilizing the CDs on filter paper substrates.

In addition to the cations, ratiometric fluorescent detections of anions are also feasible by the use of dual-emissive CDs. Both representative works were reported by Chen’s group [61,99]. Nitrite is famous as a food additive. Nevertheless, excessive intake of nitrite can cause severe diseases such as cancer or hypertension. Relying on the quenching of red emission of yellow/red dual-emissive CDs by nitrite in the mechanism of static quenching effect (SQE), ratiometric fluorescent sensing of nitrite in food samples was achieved. The sensor revealed an LOD of 31.61 nM and a wide dynamic sensing range of 0.1–100 μM. Peroxynitrite is a kind of reactive oxygen species that can destroy the important components of cells, thus causing human diseases. The authors synthesized green/red dual-emissive carbon dots, which were responsive to the peroxynitrite by showing decreased green emission whilst unchanged red emission. The energy levels alignment indicated that electron transfer from target to excited CDs was responsible for the green emission quenching. Under optimal conditions, the linear detection range was 0.03–60 μM with an estimated LOD of 11.6 nM. Moreover, ratiometric fluorescent imaging of intracellular peroxynitrite was realized with the proposed dual-emissive CDs.

### 4.3. Ratiometric Fluorescent Imaging

Biological systems are complicated by the coexistence of diverse kinds of biomolecules, salts, nutrients, and so forth. Consequently, to specifically and accurately image a target in biological media is difficult due to the interferences of, for instance, auto-fluorescent molecules, the quenching effect of concentrated salts (larger than 100 mM), etc. False-positive or negative results may be obtained for the single-emissive probes. On this occasion, dual-emissive CDs show high potential in cell imaging applications for their superiority in providing ratiometric imaging results. What should be noted is that a certain amount of the accounted ratiometric fluorescent sensors have been used to image pH or ions in model cells [51,68,98,99,100,101]. Herein, we will focus on those dual-emissive CDs-based ratiometric probes for imaging of microenvironments or biomarkers.

**Figure 9:**
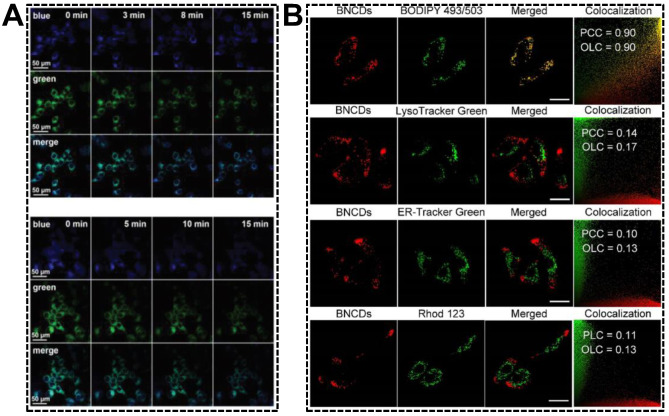
(**A**) Fluorescent images of MCF-7 cells pre-treated with dual-emissive CDs after the addition of tunicamycin (top group) and under hypoxic conditions (bottom group) for varying time. Reproduced with permission [49]. Copyright 2020, The Royal Society of Chemistry. (**B**) Fluorescent images of CDs-incubated living cells co-stained with dyes for lipid drops, lysozyme, ER, and mitochondria. Reproduced with permission [103]. Copyright 2022, Elsevier Ltd.

The relative emission intensity ratio of dual-emissive CDs carrying hydrophilic or amphiphilic groups might be sensitive to the media polarity, inspiring researchers to exploit novel strategies for water detection in the organic phase [81] or critical micelle concentration in aqueous solution [102]. Living cells are combinations of both hydrophilic media and hydrophobic subcellular structures (lipid drops, lipoproteins, lipid bilayer of membranes, etc.). Changes in the amounts of hydrophobic fractions or organelle polarity are usually accompanied by metabolic disorders. Having validated the green emission quenching of the blue/green emissive CDs in the presence of higher concentrations of polar component, as well as the endoplasmic reticulum (ER) targeting capability, Shuang et al. applied the CDs in imaging of ER polarity [49]. The variations in ER polarity were adjusted by drug (tunicamycin) stimuli and hypoxic conditions. Both resulted in the accumulation of polar proteins and the decrease in nonpolar proteins. As shown in Figure 9A, the green emission of dual-emissive CDs obviously decreased with the treatment time. In contrast, the blue emission remained almost unchanged, suggesting an increase in the polarity of the ER under ER stress. By exploiting the lipophilic CDs that can simultaneously emit orange and red fluorescence, Wu’s group reported the specific imaging of cellular lipid drops [103]. As shown in Figure 9B, the merged fluorescent images of cells indicated the good colocalization of CDs and BODIPY 493/503 (a neutral lipid drop dye). The Pearson’s correlation coefficient and overlap coefficient values of the CDs with BODIPY 493/503 were much higher than those of the CDs with the dyes for lysozyme, ER, or mitochondria. Importantly, the dual-emissive CDs were capable of effectively tracking the dynamic changes of lipid drops in living animal cells.

Based on an indicator displacement assay (IDA) principle, a Cu(II)-assisted orange/green dual-emissive CD was exploited for the ratiometric fluorescent detection and imaging of anthrax biomarker (dipicolinic acid, DPA) [104]. The green emission of dual-emissive CDs was efficiently quenched upon complexation with Cu(II). However, in the presence of DPA, the green emission was recovered due to the stronger chelation between DPA and Cu(II). Further, E. coli bacteria were used as the model to assess the performance of the CDs-Cu(II) probe in imaging intracellular DPA. Obviously, the orange channel fluorescence remained unchanged, while an “on-off-on” transition process of green channel fluorescence was observed along with the continuous addition of Cu(II) and DPA. The result demonstrated that the established CDs-Cu(II) probe was capable of monitoring DPA in bacteria.

### 4.4. Other Applications

Beyond the applications of dual-emissive CDs in photoelectric areas and analytical science, one of the most impressive works was to enhance the biological photosynthetic efficiency with dual-emissive CDs [105]. In this work, the CDs exhibited bright blue and red emissions, which exactly matched the absorption spectrum of chloroplasts. The intro experiment declared that the chloroplasts coated by the dual-emissive CDs could produce 2.8 times more ATP than the bare ones. More importantly, the photosynthesis of a living plant was also enhanced by transplanting CDs into leaves. The maximum increment in electron transport rate was determined as 25% in comparison with the leaves without CDs. As the first attempt to utilize the nontrivial dual-emissive property of CDs to elevate the plant performance in solar energy conversion, this work could be a milestone in the development of the nanobiotic area.

## 5. Conclusions and Prospects

This review summarizes the state of the art of synthesis strategies, optical mechanisms, and applications of dual-emissive CDs. The synthesis strategies can be roughly categorized as controllable carbonization, semi-reservation of precursor emission center, and heteroatom doping. The dual-emission trait is mainly attributed to the mechanisms of intra-particle electron/energy transfer and inter-particle interactions. As an ideal alternative to the multi-emissive nanosystems composed of multiple fluorophores, the “dual-emission in one-dot” CDs can be applied to fabrications of single-component WLEDs with satisfying white light emission and electroluminescence efficiency, as well as constructions of ratiometric fluorescent sensors for single target/multiplexed detections. In addition, their remarkable biocompatibility allows the dual-emissive CDs to be used for ratiometric fluorescent imaging of cellular substances.

Despite the great progress that has been made in the past few years, further research on dual-emissive CDs is still urgently needed to address the problems as follows: 1. Greener and faster synthesis approaches are needed to prepare dual-emissive CDs. As can be noticed from the second part of this review, long-time hydrothermal treatment of precursors is the prior method to prepare dual-emissive CDs. However, the large-scale synthesis of CDs requires more time-saving and cost-effective approaches, such as microwave-assisted carbonization or the possible “top-down” routes. 2. The formation mechanism of the dual-emissive CDs needs to be further clarified. The polymerization and carbonization procedure is now widely accepted to explain the formation of CDs during the synthesis. However, the detailed chemical insights behind polymerization and dehydration are far less elaborated. Up to date, limited reports corresponding to the synthesis of single-emissive CDs have analyzed the stepwise reactions, as well as the chemical and morphological details of the intermediate products during the CD formations. It is believed that the declaration of the CD formation mechanism is of great significance in accurately predicting and tuning the optical and chemical properties of dual-emissive CDs by careful precursor screening and synthesis condition controlling. 3. The emission mechanism of the dual-emissive CDs is yet debatable. Presently, emissions located at different wavelengths are claimed to be stemmed from intra-particle electron/energy coupling or inter-particle interactions. Nevertheless, a significant number of papers just speculated the emission mechanism and energy levels of different emission centers are rarely depicted. The difficulties in defining the boundary between sp^3^ and sp^2^-hybridized carbon atoms and resolving the structures of molecular (functional groups) emission centers are the main obstacles to the in-depth study of the exact emission mechanisms.

It is anticipated that by harnessing high-end characterization techniques, these problems can be well addressed in the near future. At the time, more diverse synthesis approaches, definite emission mechanisms, and more purposeful tuning of morphological and optical properties of dual-emissive CDs will emerge. Based on this, more extensive and practical applications of dual-emissive CDs must be seen.

## Data Availability

Not applicable.
